# Fufang Xue Shuan Tong capsules inhibit renal oxidative stress markers and indices of nephropathy in diabetic rats

**DOI:** 10.3892/etm.2012.680

**Published:** 2012-08-23

**Authors:** DONGHONG FANG, XUESI WAN, WANPING DENG, HONGYU GUAN, WEIJIAN KE, HAIPENG XIAO, YANBING LI

**Affiliations:** Department of Endocrinology and Diabetes Center, The First Affiliated Hospital of Sun Yat-sen University, Guangzhou, Guangdong 510080, P.R. China

**Keywords:** Fufang Xue Shuan Tong capsule, diabetic nephropathy, oxidative stress, malondialdehyde, superoxide dismutase

## Abstract

Fufang Xue Shuan Tong (FXST) capsules, a traditional Chinese medicine, have been used to treat diabetic nephropathy for many years. FXST has been shown to attenuate elevated levels of oxidative stress in the retina of diabetic rats. However, whether FXST protects kidneys through the same mechanism(s) remains unclear. In this study, diabetes was induced in rats by administration of a high-fat diet and low-dose streptozotocin. Rats were administered low (450 mg/kg/day), middle (900 mg/kg/day) or high (1800 mg/kg/day) doses of FXST orally for 3 months. Another group was administered 50 mg/kg/day orally for the same period. The results indicated that all doses of FXST reduced urinary protein excretion and creatinine clearance and ameliorated the diabetic nephropathy-related mesangial matrix expansion. However, only middle and high doses of FXST prevented glomerular hypertrophy in diabetic rats, and the high dose showed the greatest inhibitory effect with regard to mesangial matrix expansion. Furthermore, superoxide dismutase activities were significantly elevated, whereas malondialdehyde levels were significantly reduced in the renal cortex following FXST treatment. The kidney-protective role of FXST is not inferior to that of captopril, one of the most commonly used drugs for the treatment of diabetic nephropathy. In conclusion, FXST retards the progression of diabetic nephropathy, while high-dose FXST shows the most prominent effect in counteracting the pathological changes of diabetic nephropathy. The renoprotective action of FXST is induced by the reduction of oxidative stress in diabetic nephropathy.

## Introduction

Diabetic nephropathy is a major microvascular complication of diabetes mellitus and the leading cause of end-stage renal disease (1). The interventions in general clinical use are not capable of efficiently slowing or reversing the progression of nephropathy. Therefore, interventions which could optimally delay the development of diabetic nephropathy are required.

The traditional Chinese medicine Fufang Xue Shuan Tong (FXST) capsule, which contains SanQi, DanShen, XuanShen and HuangQi, has been used to treat a series of fundus oculi diseases, including diabetic retinopathy, for many years in China (2,3). Since microvascular diseases share part of the same pathogenesis, we hypothesized that FXST may also exhibit a nephropathy-protective effect in diabetes.

Oxidative stress is increased in patients with diabetes and in various tissue samples from experimental diabetes (4–6). Accumulating evidence has shown that oxidative stress markers, such as malondialdehyde (MDA) and 8-hydroxy-2′-deoxyguanosine (8-OHdG) are increased in diabetic nephropathy states. Antioxidant enzymes, such as superoxide dismutase (SOD), catalase and glutathione peroxidase, exhibit a relatively low expression. Accordingly, oxidative stress is a significant contributor to the pathogenesis of diabetic nephropathy, and therefore those interventions with anti-oxidant properties may attenuate the manifestations associated with diabetic nephropathy (7,8). FXST was observed to attenuate the up-regulation of oxidative stress in an experimental model of diabetic retinopathy (3). Since oxidative stress is a common cause of diabetic retinopathy and nephropathy (9), we further hypothesized that the nephropathy-protective effect of FXST was also mediated by the down-regulation of oxidative stress. However, the precise effects and mechanisms still need to be addressed using cellular and molecular approaches.

To test these hypotheses, diabetes was induced in rats by administration of a high-fat diet and low dose streptozotocin (STZ) in the current study. FXST was initiated in three different doses to assess the effects of FXST on diabetic nephropathy and the potential causal mechanisms.

## Materials and methods

### Animal model

A total of 59 male Sprague-Dawley (SD) rats (150–180 g) were purchased from the Experimental Animal Center of Guangdong Medical Sciences and were raised in the Department of Laboratory animal center of Sun Yat-sen University. We were unable to induce diabetes in 9 and 7 died during the study, therefore 43 rats completed the study. The study followed the Guidelines for Animal Care issued by the First Affiliated Hospital of Sun Yat-sen University.

The rats were allocated a normal or high-fat diet (HFD) (58.3% fat, 7.9% protein and 33.8% carbohydrate, as a percentage of total kcal) *ad libitum*, respectively, for 5 weeks. Diabetes was induced in HFD rats by intraperitoneal injection of STZ (Sigma, St. Louis, MI, USA), 40 mg/kg body weight. The normal-diet rats were injected with an equal volume of vehicle citrate buffer. Three days after STZ injection, the rats with a non-fasting blood glucose of ≥16.7 mmol/l were considered diabetic and selected for additional studies.

### Experimental protocol

Diabetic rats were randomized into five groups: Low-dose FXST group: LF group, n=7, FXST 450 mg/kg/day, oral gavage; middle-dose FXST group: MF group, n=6, FXST 900 mg/kg/day, oral gavage; high-dose FXST group: HF group, n=7, FXST 1800 mg/kg/per day, oral gavage; captopril group: CA group, n=7, captopril 50 mg/kg/day, oral gavage; diabetic group: DM group, n=8, no treatment. Normal-diet non-diabetic SD rats served as controls (NC group, n=8).

The treatment was initiated 3 weeks after the induction of diabetes. After 3 months of treatment, the rats were fasted overnight and anesthetized by intraperitoneal injection of 10% chloral hydrate (0.3 ml/100 g body weight). The right kidneys were removed for histological analysis. The left kidneys were removed, decapsulated, weighed and then divided into cortical and medullary sections. Kidney cortices were snap-frozen in liquid nitrogen and stored at −80°C for further analysis.

### Biochemical analysis

Fasting blood samples were obtained from the tail veins. Blood glucose was measured by glucometer (Roche, Basel, Switzerland). Serum and urine levels of creatinine were detected using an automatic biochemistry analyzer. Rats were kept in metabolic cages to collect 24-h urine. Susequently, the urine volume was measured and urine protein was tested by chemiluminescence analysis. Urinary protein excretion (mg/24 h) was assessed as: urine protein (mg/l) x urine volume (liters)/24 h. Creatinine clearance rate (Ccr) was determined as: Ccr= urine creatinine (μmol/l) x urine volume per min (ml/min)/serum creatinine (μmol/l) and data were normalized for body weight.

### Histological analysis

The right kidneys were fixed in 10% buffered formalin, embedded in paraffin, sectioned at 4 μm and stained with hematoxylin and eosin and periodic acid-silver metheramine (PASM). In PASM-stained sections, the glomerular cross-sectional (Ag), tuft (At) and mesangial matrix (Am) areas were measured in 30 glomerular profiles per rat using Image Pro Express 6.0 software. The mesangial matrix area was defined as the PASM-positive area. Quantitative measurement of mesangial matrix expansion (mesangial matrix index) was expressed as the PASM-positive area per total glomerular tuft cross-sectional area (10). The glomerular volume (Vg) was determined as: Vg = β/κ [Ag]3/2, where β is 1.38 as a shape factor and κ is 1.1 as a distribution factor (11).

### Measurement of lipid peroxidation

Kidney cortices (100 mg) were weighed and homogenized. The protein concentration was determined by BCA analysis (Kangchen, Shanghai, China). Lipid peroxidation, measured as MDA, reflects the impact of oxidative stress in tissues. Tissue MDA levels were measured according to the method described by Ohkawa *et al* with a commercially available kit, following the manufacturer’s instructions (Genmed, Shanghai, China) (12). The absorbance was measured with a spectrophotometer at 535 nm. MDA levels are expressed as MDA (μmol)/protein (μg).

### SOD

SOD activity was assayed using the nitroblue tetrazolium (NBT) method with a commercial assay kit according to the manufacturer’s instructions (Genmed, Shanghai, China) (13). The absorbance was measured with a spectrophotometer at 560 nm. One unit (U) of SOD is defined as the amount of protein that inhibits the rate of NBT reduction by 50%. The calculated SOD activity is expressed as SOD (U)/protein (μg).

### Statistical analysis

Results were shown as the means ± SD. Statistical analysis was performed using the SPSS 11.0 statistical package. One-way-analysis of variance (one-way-ANOVA) was used for comparison of more than two groups followed by an LSD test for multiple comparisons. The Kruskal-Wallis test was used when the data departed substantially from a normal distribution. Significance was defined as p<0.05.

## Results

### Metabolic data

The levels of fasting blood glucose were significantly increased in the DM, LF, MF, HF and CA groups prior to intervention and remained higher for the entire duration, as compared with the NC group. Body weight showed the inverse result. Three weeks after the induction of diabetes, the body weights were significantly decreased and remained lower over the treatment period in the diabetic rats with or without treatment, in comparison to the NC group. The fasting blood glucose and body weights in the DM and treatment groups did not reach statistical significance (Table I).

### Urinary protein excretion

**Figure 1:**
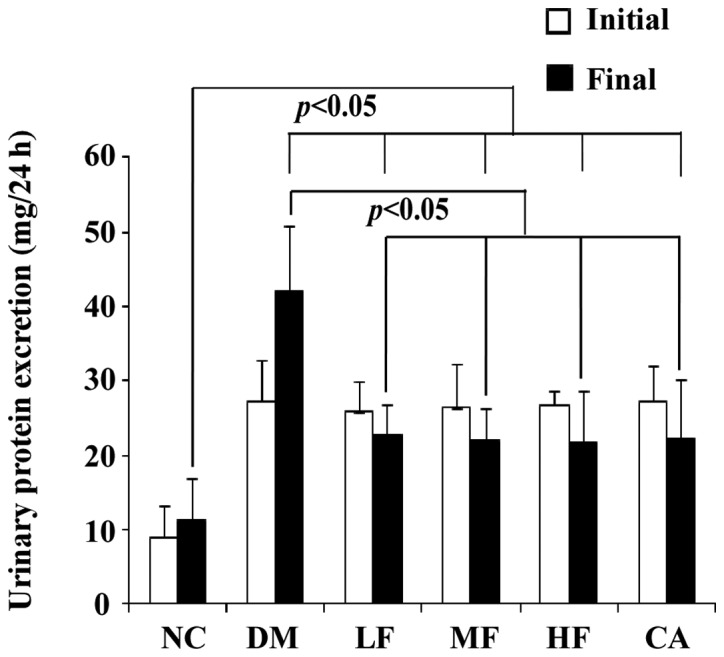
Urinary protein excretion is shown. Induction of 3 weeks of diabetes led to a marked increase in urinary protein excretions (p<0.05). Three months of FXST and captopril therapy significantly decreased the high levels of urinary protein excretion in diabetic rats (p<0.05). However, the levels in the treatment groups remained higher than those in the NC group after 3 months of intervention (p<0.05).

Urinary protein excretion was already significantly elevated after 3 weeks of diabetes and markedly declined after 3 months of FXST and captopril treatments as compared to the no-treatment DM group. However, urinary protein excretion in the treatment groups remained higher than that in the NC group. The differences of urinary protein excretion among the various doses of FXST and captopril groups were not significant (Fig. 1).

### Creatinine clearance

Creatinine clearance remained higher 3 weeks after induction of diabetes and averaged at lower levels after 3 months of FXST and captopril therapy, albeit it remained largely unchanged in the DM group. Creatinine clearances in the LF, MF, HF and CA groups were higher than those in the NC group, although they did not reach statistical significance. The amelioration of creatinine clearance in the intervention groups remained significant when normalized for body weight. Moreover, when normalized for body weight, creatinine clearance in the DM group was reduced, with the most likely reason being that rats gained weight over the study period (Table II). Following 3-month FXST treatment, urinary protein excretion and creatinine clearance markedly decreased, but remained slightly higher than the normal levels, indicating that FXST was capable of delaying but not completely reversing disease progression.

### Kidney weight and relative kidney weight

**Figure 2:**
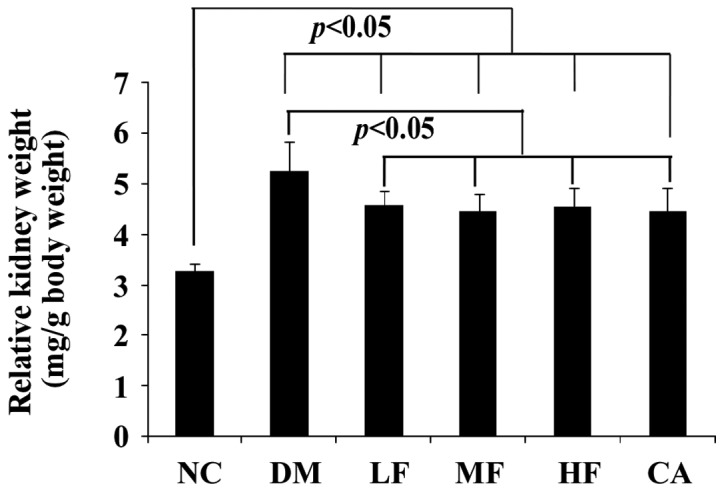
Kidney weight and relative kidney weight. The relative kidney weights were reduced in the treatment groups and remained higher in the DM group. The NC group showed the lowest relative kidney weight.

Induction of diabetes significantly increased the kidney weight and relative kidney weight. The changes were highly suppressed in the LF, MF, HF and CA groups and did not reach statistical significance. However, the relative kidney weight in the NC group remained lower than that in the treatment groups. The potential reason was that the diabetic rats did not gain weight as significantly as the normal rats during the study (Table III, Fig. 2).

### Histological analysis

**Figure 3:**
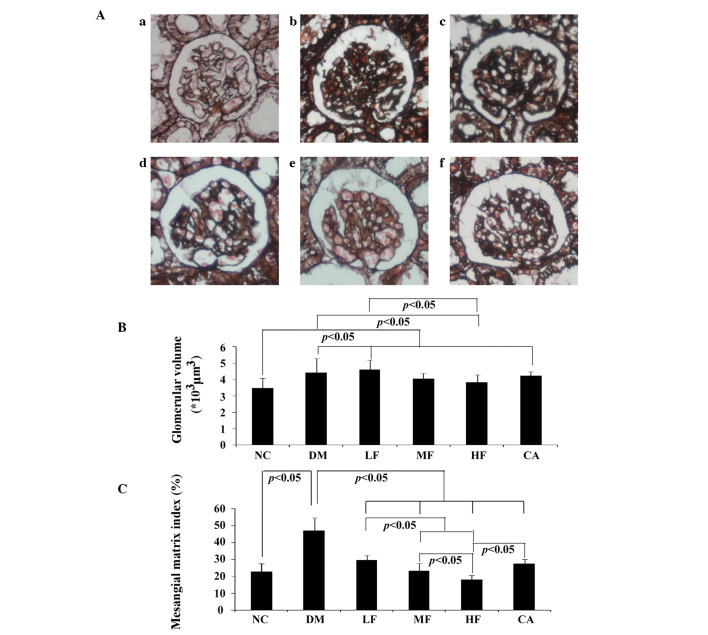
PASM staining sections of the kidneys, the glomerular volume (Vg) and mesangial matrix index. (Aa) NC group; (Ab) DM group; (Ac) LF group; (Ad) MF group; (Ae) HF group; and (Af) CA group. Original magnification for Aa–f was x400. (B and C) Three-month FXST or captopril treatment abrogated mesangial matrix expansion in diabetic rats, and the HF group showed the most pronounced effect. However, only the middle and high dose of FXST suppressed glomerular hypertrophy. Significant diabetic glomerulosclerosis was not observed in any rat kidney.

PASM-stained glomeruli are representatively shown in Fig. 3Aa–f. Glomerular hypertrophy and mesangial matrix expansion, measured as glomerular volume and mesangial matrix index respectively, were markedly elevated in the DM group. Mesangial matrix expansion was attenuated in the treatment groups after 3-month FXST or captopril therapy, and the HF group showed the most prominent effect in antagonizing mesangial matrix expansion. By contrast, glomerular hypertrophy was ameliorated only in the MF and HF groups, but not in the LF or CA groups. Significant diabetic glomerulosclerosis was not observed in any rat kidney (Fig. 3B and C).

### MDA

**Figure 4:**
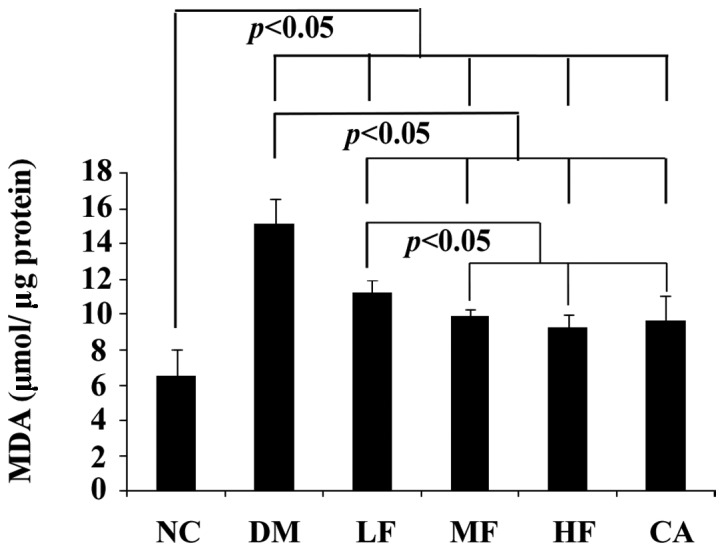
FXST reduced renal cortical MDA. In comparison to the DM group, the MDA levels in the LF, MF, HF and CA groups were markedly reduced. The LF group showed the highest level in the treatment groups.

A marked decrease was detected in the levels of renal cortical MDA in the LF, MF, HF and CA groups as compared with the DM group, showing that FXST and captopril reduced the oxidative status. However, in comparison to the NC group, the levels in these groups remained higher. Furthermore, the finding that the decrease in the LF group was less prominent than that in the MF and HF group indicated that the antioxidative effect of FXST was dose-dependent (Fig. 4).

### SOD

**Figure 5:**
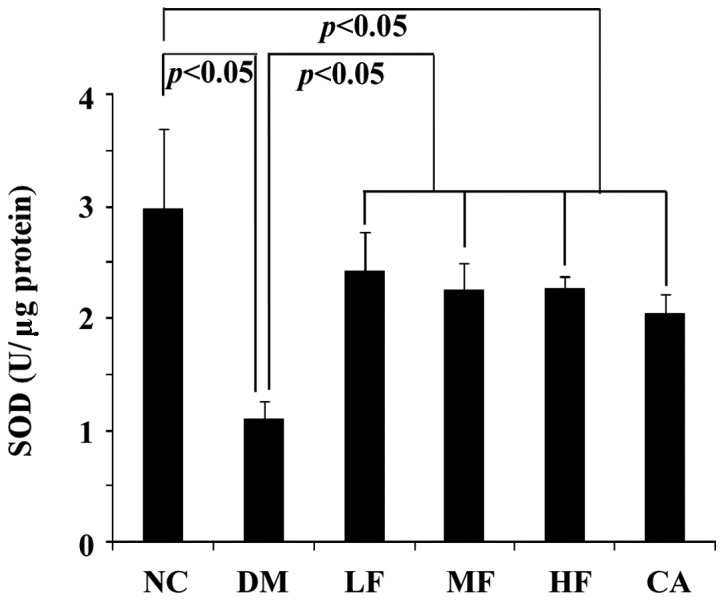
FXST elevated renal cortical SOD. All doses of FSXT and captopril notably increased the levels of renal cortical SOD as compared with the DM group, but remained lower than the NC group.

The levels of renal cortical SOD were significantly increased in the LF, MF, HF and CA groups as compared with the DM group, but remained lower than those in the NC group. Captopril showed the most pronounced effect in activating the antioxidant system. However, the difference did not reach statistical significance (Fig. 5).

## Discussion

The major findings of the current study are that FXST decreases urinary protein excretion, reduces creatinine clearance and ameliorates the diabetic nephropathy-related histopathological changes. FXST retards the progression of diabetic nephropathy through modulations of oxidative stress. The beneficial effects of FXST have been shown to be similar to those of captopril.

In its normal state, the kidney generates a substantial amount of oxidative stress due to its high metabolic activity, which is balanced by an extensive antioxidant system. However, under pathological conditions such as diabetes, oxidative stress balance shifts towards a pro-oxidant state that accelerates tissue injury, and the kidney has been shown to be a target of oxidative stress-mediated tissue damage (14). As in our study, induction of diabetes resulted in a marked increase in the SOD levels in the renal cortex of diabetic rats. The MDA levels were also found to be significantly reduced. Therefore, oxidative stress is a potential mechanism for diabetic nephropathy, since it promotes the formation of lipid peroxidation products and decreases the antioxidant defense by decreasing the level of antioxidant enzymes. Accordingly, therapeutic strategies with anti-oxidant properties may eliminate the manifestations associated with diabetic nephropathy. In our study, FXST down-regulated the level of oxidative stress in the renal cortices of diabetic rats. Therefore, FXST may be a novel strategy with which to slow the progression of renal disease.

One of the components of FXST, SanQi, attenuated the high level of oxidative stress in the rat liver in a model of alcoholic fatty liver disease and showed an anti-oxidative effect in the serum of diabetic rats (15,16). Another component of FXST, Danshen, improved antioxidation of the patients with acute coronary syndromes following percutaneous transluminal coronary intervention (17). Danshen reduced the level of MDA and enhanced the level of SOD in cerebral tissues in a focal cerebral ischemia rat model (18). The third component of FXST, Huangqi, elevated the activity of SOD and reduced MDA in patients with primary nephrotic syndrome (19). In an *in vivo* study, Huangqi antagonized hydrogen peroxide (H_2_O_2_)-induced oxidative injury in cardiomyocytes (20). Polyphenols in Xuanshen, the fourth ingredient of FXST, also possess high antioxidant activity (21). Therefore, the anti-oxidant effect of FXST may be due to the anti-oxidant properties of all of its active ingredients. These ingredients, when combined together, exhibit optimal activity in anti-oxidative stress (3).

Under normal circumstances, oxidative stress is counteracted by antioxidant enzymes such as SOD, which normally scavenges superoxide. The role of SOD is crucial to the regulation of oxidative stress in diabetes. Enhanced activity of antioxidant enzymes has been reported as an adaptive mechanism to protect cells against the toxicity of free radicals. The decreased SOD in the untreated diabetic rats may indicate that the protective ability in response to elevated levels of oxidative stress was impaired. Increased SOD activity induced by FXST may be a response to increased generation of superoxide anions in diabetes and may therefore result in the amelioration of oxidative stress. MDA, an end-product of lipid peroxidation and a measure of free radical generation, reflects the impact of oxidative stress in cells and tissues. In the present study, renal cortical MDA concentrations in diabetic rats were significantly elevated. This is consistent with previously studies. The increased MDA levels indicate the occurrence of lipid oxidative damage, which is suggested in the development of diabetic nephropathy. Treatment with FXST significantly reduced the levels of MDA. These results indicate that FXST exhibits an anti-peroxidative effect. Taken together, our results have shown that SOD activity increased, whereas MDA activity was reduced in the renal cortex of FXST-treatment diabetic rats.

Oxidative stress is well known as an important factor in the progression of diabetic complications, is involved in molecular changes associated with exacerbation of renal injury, and results in mesangial expansion and increased extracellular matrix deposition (22). Therefore, as shown in our study, the anti-oxidant activity of FXST eliminated the pathological abnormalities of diabetic nephropathy, thereby reducing urinary protein excretion, which was a marker for the development of nephropathy in diabetes. Therefore, FXST may arrest the progression of renal disease. Moreover, the renoprotective activity of FXST was mediated by modulation of oxidative stress in diabetic nephropathy.

In conclusion, Fufang Xue Shuan Tong capsule, a traditional Chinese medicine, delayed the development of proteinuria, reduced creatinine clearance, attenuated the pathological abnormalities of diabetic nephropathy, thereby showing predominant kidney-protective action. This finding may be attributable to the fact that the increased oxidative stress in the kidney cortex of diabetic rats was antagonized by FXST. The protective role of FXST is not inferior to that of captopril, one of most commonly used drugs for the treatment of diabetic nephropathy.

## Figures and Tables

**Table I:** Metabolic data.

		Blood glucose (mmol/l)		Body weight (g)	
Group	n	Initial	Final	Initial	Final
NC	8	4.99±0.58	4.99±0.84	412.50±22.96	547.75±34.50
DM	8	21.04±5.96^a^	23.29±2.88^a^	286.75±59.17^a^	389.88±25.06^a^
LF	7	19.56±4.94^a^	21.70±5.97^a^	255.85±20.88^a^	386.71±28.04^a^
MF	6	20.87±5.24^a^	22.42±3.83^a^	258.66±10.46^a^	394.33±26.43^a^
HF	7	19.09±2.82^a^	22.27±3.65^a^	257.14±18.08^a^	383.71±31.54^a^
CA	7	21.24±3.76^a^	22.76±4.97^a^	267.85±27.15^a^	386.28±39.92^a^

a p<0.05 vs. NC group;

b p<0.05 vs. DM group. Compared with those in the NC group, the values of fasting blood glucose were significantly increased and the body weights were markedly decreased in the DM, LF, MF, HF and CA groups prior and subsequent to intervention, but did not reach statistical significance among the groups. NC, normal-diet controls; DM, diabetic group; LF, low-dose FXST group; MF, middle-dose FXST group; HF, high-dose FXST group; CA, captopril group.

**Table II:** Creatinine clearance.

		Creatinine clearance (ml/min)		Relative creatinine clearance (ml/min/100 g body weight)	
Group	n	Initial	Final	Initial	Final
NC	8	1.28±0.42	1.43±0.92	0.31±0.10	0.26±0.17
DM	8	3.89±1.51^a^	3.87±0.74^a^	1.37±0.48^a^	0.99±0.18^a^
LF	7	3.64±1.65^a^	2.04±1.13^b^	1.43±0.67^a^	0.54±0.32^b^
MF	6	3.49±0.84^a^	1.93±1.48^b^	1.35±0.30^a^	0.50±0.39^b^
HF	7	3.51±0.55^a^	1.82±1.19^b^	1.38±0.28^a^	0.49±0.36^b^
CA	7	3.47±1.00^a^	1.81±0.74^b^	1.32±0.42^a^	0.48±0.23^b^

a p<0.05 vs. NC group;

b p<0.05 vs. DM group. Creatinine clearance was increased 3 weeks after the induction of diabetes and averaged at lower levels after 3 months of FXST and captopril therapy but remained almost unchanged in the DM group. Creatinine clearances in the LF, MF, HF and CA groups were slightly higher than those in the NC group, but did not reach statistical significance. When normalized for body weight, creatinine clearance in the treatment groups was also lower than that in the DM group. NC, normal-diet controls; DM, diabetic group; LF, low-dose FXST group; MF, middle-dose FXST group; HF, high-dose FXST group; CA, captopril group.

**Table III:** Kidney weight and relative kidney weight.

Group	n	Body weight (g)	Kidney weight (g)	Relative kidney weight (mg/g body weight)
NC	8	547.75±34.50	1.79±0.10	3.28±0.15
DM	8	389.88±25.06^a^	2.05±0.15^a^	5.27±0.56^a^
LF	7	386.71±28.04^a^	1.76±0.03^b^	4.58±0.29^a,b^
MF	6	394.33±26.43^a^	1.75±0.03^b^	4.46±0.34^a,b^
HF	7	383.71±31.54^a^	1.73±0.03^b^	4.55±0.38^a,b^
CA	7	386.28±39.92^a^	1.71±0.02^b^	4.47±0.45^a,b^

a p<0.05 vs. NC group;

b p<0.05 vs. DM group. The kidney weight and relative kidney weight were notably increased in the diabetic rats. The changes were highly suppressed in the LF, MF, HF and CA groups and did not reach statistical significance in these groups. However, the relative kidney weight in the NC group remained lower than that in the treatment groups. NC, normal-diet controls; DM, diabetic group; LF, low-dose FXST group; MF, middle-dose FXST group; HF, high-dose FXST group and CA, captopril group.
